# Filovirus-reactive antibodies in humans and bats in Northeast India imply zoonotic spillover

**DOI:** 10.1371/journal.pntd.0007733

**Published:** 2019-10-31

**Authors:** Pilot Dovih, Eric D. Laing, Yihui Chen, Dolyce H. W. Low, B. R. Ansil, Xinglou Yang, Zhengli Shi, Christopher C. Broder, Gavin J. D. Smith, Martin Linster, Uma Ramakrishnan, Ian H. Mendenhall

**Affiliations:** 1 National Centre for Biological Sciences, Tata Institute of Fundamental Research, Bangalore, India; 2 Sastra University, School of Chemistry and Biotechnology, Thanjavur, Tamil Nadu, India; 3 Uniformed Services University of the Health Sciences, Department of Microbiology and Immunology, Bethesda, Maryland, United States of America; 4 Duke-National University of Singapore Medical School, Programme in Emerging Infectious Diseases, Singapore; 5 National University of Singapore, Graduate School for Integrative Sciences and Engineering, Singapore; 6 Manipal Academy of Higher Education, Manipal, Karnataka, India; 7 Wuhan Institute of Virology, Department of Emerging Infectious Diseases, Wuhan, China; Center for Disease Control and Prevention, UNITED STATES

## Abstract

Bats are reservoirs for several zoonotic pathogens, including filoviruses. Recent work highlights the diversity of bat borne filoviruses in Asia. High risk activities at the bat-human interface pose the threat of zoonotic virus transmission. We present evidence for prior exposure of bat harvesters and two resident fruit bat species to filovirus surface glycoproteins by screening sera in a multiplexed serological assay. Antibodies reactive to two antigenically distinct filoviruses were detected in human sera and to three individual filoviruses in bats in remote Northeast India. Sera obtained from *Eonycteris spelaea* bats showed similar patterns of cross-reactivity as human samples, suggesting them as the species responsible for the spillover. In contrast, sera from *Rousettus leschenaultii* bats reacted to two different virus glycoproteins. Our results indicate circulation of several filoviruses in bats and the possibility for filovirus transmission from bats to humans.

## Author summary

Focused virus surveillance at human-wildlife interfaces enables proactive detection of potentially epidemic pathogens. Filoviruses, including ebolaviruses and marburgviruses, are pathogens with epidemic potential. They were previously detected in bats and have caused disease outbreaks in humans with a high case fatality rate. Here, we tested sera obtained from bats and humans at a high-risk interface for the presence of filovirus reactive antibodies. Human participants were engaged in annual bat hunts, possibly exposing them to bat-borne viruses. We report the exposure of humans to filoviruses that were likely derived from the two sampled bat species. The bats contain antibodies raised to presumably three distinct filoviruses. Our findings suggest bats in South Asia act as a reservoir host of a diverse range of filoviruses and filovirus spillover occurs through human exposure to these bats.

## Introduction

Filoviruses are causative agents of viral haemorrhagic disease in humans and non-human primates although virus spillover is rare [1]. There are ten distinct filoviruses classified into four genera, *Ebolavirus*: Ebola virus, Bundibugyo virus, Taï forest virus, Sudan virus, Reston virus and Bombali virus; *Marburgvirus*: Marburg virus and Ravn virus; *Cuevavirus*: Lloviu virus; and *Dianlovirus*: Měnglà virus [2–4].

Bats are the proposed natural reservoir of filoviruses, involved in enzootic virus maintenance and zoonotic virus transmission to susceptible hosts [4]. The majority of described filoviruses are endemic in the African continent, although filovirus-specific antibodies were detected in bats from Bangladesh [5], the Philippines [6], and Singapore [7]. The genome of a novel filovirus, Měnglà virus, was detected in bats from China [8] and is the second Asiatic filovirus described after Reston virus [9]. Lloviu virus was discovered in Spain in 2011 and detected in Hungary in 2016 [10, 11]. Bats are hunted by humans across Africa and Asia, and at least 167 bat species are consumed [12]. High-risk activities, such as bat hunting and mining in bat-dwelling caves, pose a threat of cross-species filovirus transmission [13].

In the Northeast Indian state of Nagaland, local ethnic groups have conducted bat harvests for at least seven generations as a source of food and traditional medicine. These bat hunters are exposed to saliva, blood, and excreta from the bat species *Rousettus leschenaultii* and *Eonycteris spelaea*. We conducted a serological survey of both hunted bat species and human hunters to study if humans have been exposed to filoviruses potentially originating from bats.

## Materials and methods

### Ethics statement

All study participants provided written informed consent by signing a form in their native language Nagamese. All human samples and surveys were collected under National Centre of Biological Sciences (NCBS) IEC permit 7/001 and National University of Singapore (NUS) IRB permit N-17-034E. Negative control sera were collected under the NUS IRB permit number H-18-029. All bats were sampled under the NCBS, Tata Institute of Fundamental Research IACUC permit #UR-6/2014, which adheres to provisions of the Prevention of Cruelty to Animals Act (1960) and the Breeding of and Experiments on Animals Rules (1998) and the NUS IACUC permit B16-0159 under the National Advisory Committee for Laboratory Animal Research (NACLAR) guidelines in Singapore.

### Sample and data collection

**Fig 1:**
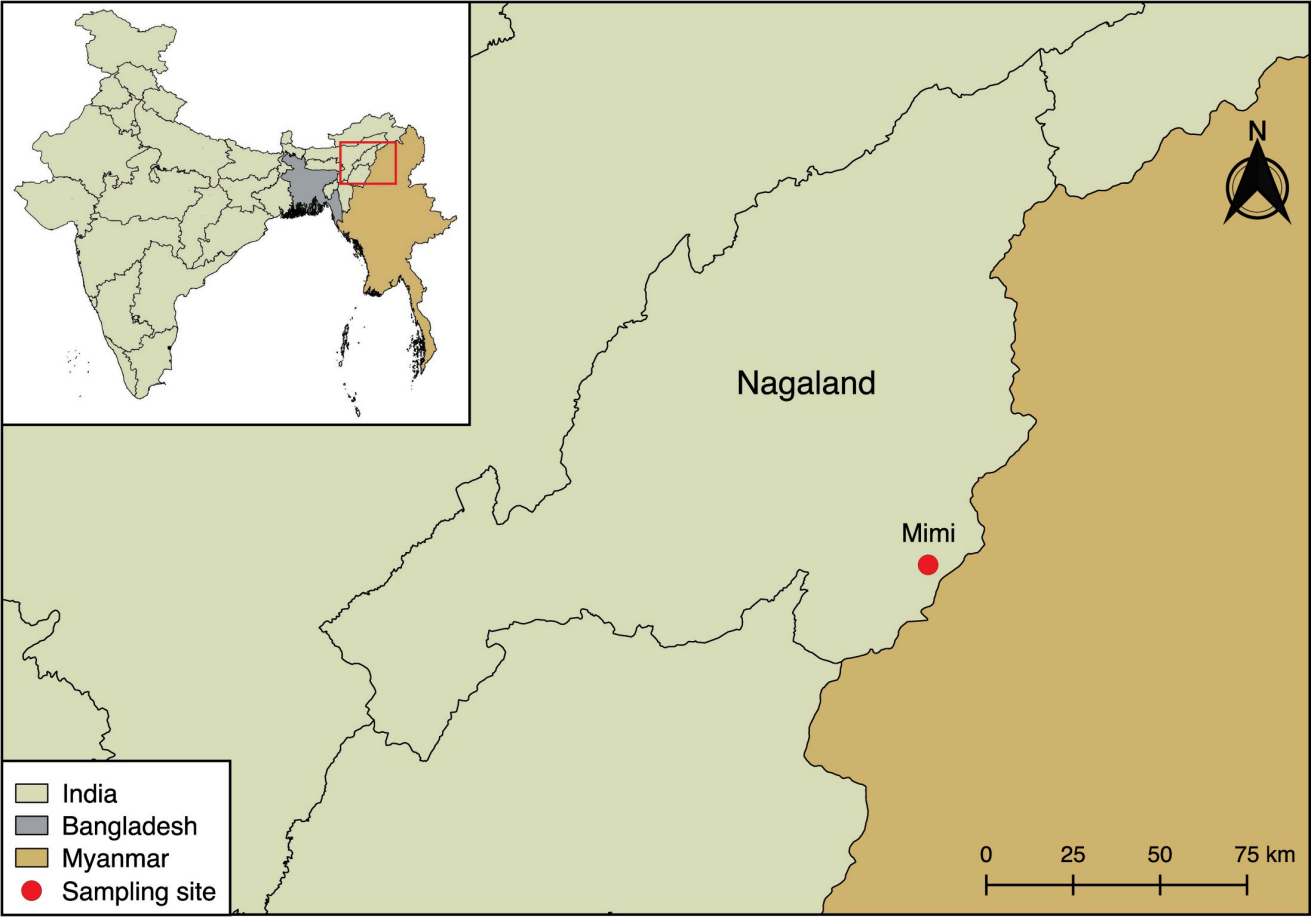
Geographical map of the border region between India and Myanmar. The Indian state of Nagaland and Mimi village are indicated. The map was created using QGIS v2.18.7 software (https://qgis.org/en/site/). The India shapefile was downloaded from the India Remote Sensing and GIS website (http://www.indianremotesensing.com/) and the Bangladesh and Myanmar shapefiles were downloaded from DIVA-GIS (http://www.diva-gis.org/gdata). All layers were in the geographic coordinate system WGS 84 and all software and map layers used are open access.

In 2017, 85 individuals participating in an annual bat harvest in Mimi village (Fig 1) were provided a paper-based survey to record their gender, age, occupation and number of times involved in the bat harvest. Blood of consenting volunteers was collected in a serum separation tube (Vacutainer, Becton Dickinson, New Jersey, USA). Bat blood from *E*. *spelaea* (n = 16) and *R*. *leschenaultii* (n = 30) was collected by cardiac puncture after being sacrificed by the harvesters.

Blood samples were centrifuged at 1,500 x g for 10 min and sera were stored at 4°C until transport to NCBS, where they were held at -80°C. All sera were gamma-irradiated at 20KGy with the Blood Irradiator 2000 (BRIT, Mumbai, India) and heat-inactivated at 56°C for 60 min prior to screening. Pooled kidney, lung and spleen samples obtained from *E*. *spelaea* (n = 34) and *R*. *leschenaultii* (n = 69) were collected and stored individually in RNA*Later* (Sigma-Aldrich). Personnel handling potentially infectious material in the field wore N95 particulate respirators, surgical gowns, face-shields and were double-gloved. Surfaces were disinfected with 3% Virkon solution. Needles and scalpel blades were single use and disposed in sharps containers. Sealed containers were autoclaved at the Healthcare and Research Center of the Naga Hospital Authority Kohima, Nagaland, India. Small amounts of tissue (lung, spleen, kidney) was excised, combined by individual bat and homogenized in AVL Buffer (Qiagen) at NCBS. An aliquot of homogenate was used for downstream PCR analysis, while another aliquot of homogenate was used for NGS analysis.

### PCR, next generation sequencing and serology

RNA was extracted with PureLink RNA Mini Kit (Invitrogen), and cDNA was synthesized using SuperScript III Reverse Transcriptase (Invitrogen). Samples were tested with a nested pan-filovirus PCR assay targeting the L gene as reported previously [14]. The positive control for the PCR was synthesized by Integrated DNA Technologies based on a region of the L gene of Bundibugyo virus (Genbank Accession: KU182911). The plasmid was amplified and extracted after transformation into competent cells using a NucleoBond Xtra Maxi Plus EF kit (Macherey-Nagel, Düren, Germany) and following the manufacturer’s instruction. Purified plasmid was quantified, 10-fold serially diluted and validated with primers prior to use in the assay. Aliquots of homogenate for NGS analysis were further pooled by species (*E*. *spelaea* n = 34 and *R*. *leschenaultii* n = 34). NGS libraries were made (S1 Appendix) and validated by bioanalyzer, then sequenced on a HiSeqX Illumina machine with 2 x 150 bp reads by Medgenome Labs Ltd. (Bangalore, India) [15,16].

Human and bat sera samples were screened in a filovirus multiplex microsphere immunoassay as previously described [17]. Recombinant ectodomains of envelope attachment glycoproteins (GPe) from Ebola virus (EBOV), Bundibugyo virus (BDBV), Taï forest virus (TAFV), Sudan virus (SUDV), Reston virus (RESTV), Marburg virus (MARV), Ravn virus (RAVV), Lloviu virus (LLOV) and Měnglà virus (MLAV) (Table 1) were expressed in a mammalian cell-culture system [18, 19]. In 2018, purified, oligomeric GPe antigens (minus MLAV) were coupled to MagPlex microspheres (Luminex, Austin, TX, USA) and bat and human samples were diluted at 1:100 in PBS and run on a Bio-Plex 200 system (Bio-Rad, Hercules, California, USA) in duplicate. After sera incubation with GPe-coupled microspheres, samples were washed, incubated with biotinylated-Protein A and biotinylated Protein G (1:1 ratio) (Thermo Fisher Scientific, Waltham, MA, USA), washed and then finally incubated with streptavidin-phycoerythrin (PE) (Bio-Rad). After the discovery of Měnglà virus, the serum samples were re-run in 2019 with all GPe antigens and individual serum samples were diluted 1:100 for human and 1:250 for bat sera in PBS. Median fluorescence intensities (MFI) were measured using a MAGPIX machine (Bio-Rad) (S1, S2 and S3 Tables). Cell culture supernatant from a GPe untransfected cell line, was prepared and included in the multiplex immunoassay as a mock antigen sample to normalize non-specific antisera reactivity. Due to the absence of negative sera from the study site, we obtained seven presumptively negative human sera samples from a sample bank at Duke-NUS Medical School, Singapore. These were tested using a MAGPIX machine following the technical details described above in eight technical replicates to determine the variation of individual samples in repeat measurements to individual GPe (S4 Table).

**Table 1:** Virus name, host and location of isolation, and accession numbers for recombinant filovirus attachment glycoproteins (GPe) used in multiplex serological binding assays.

Virus isolate	Host/Location	Accession no.
Ebola virus/H.sapiens/COD/1976/Yambuku-Mayinga	Human/DRC	NC_002549.1
Bundibugyo virus/H. sapiens/UGA/2007	Human/Uganda	FJ217161.1
Taï Forest virus/H. sapiens/COV/1994/Pauleoula-CI	Human/Côte d'Ivoire	NC_014372
Sudan virus/H. sapiens/UGA/2000/Gulu-808892	Human/Uganda	NC_006432.1
Reston virus/M. fascicularis/USA/1989/Pennsylvania	Macaque/USA	AF522874.1
Lloviu virus/M.schreibersii-wt/ESP/2003/Asturias-Bat86	Bat/Spain	NC_016144.1
Měnglà virus/R. leschenaultii/CHN/2015/Sharen-Bat9447-1	Bat/China	KX371887.2
Marburg virus/H. sapiens/KEN/1980/Musoke	Human/Kenya	Z12132 S55429
Ravn virus/H. sapiens/KEN/1987/Kitum cave-810040	Human/Kenya	NC_024781.1

### Phylogenetic and statistical analysis

Next generation sequencing data was analysed as previously described [20]. Briefly, FASTQ files were trimmed for quality at a PHRED score of 20 and were then analysed in DIAMOND using the NCBI nr reference database [21]. DIAMOND outputs were analysed in MEGAN to determine the sequence similarly [22]. In the absence of negative serum controls from Nagaland, two independent methods were employed to define positive and negative cut-offs. The MFI values of the mock antigen were subtracted from each GPe MFI and the values were transformed to be positive, with the lowest number being 1. A log-normal model was fitted to the MFI data and the parameters of the fit were estimated in order to calculate the 95^th^ percentile of the log-normal distribution (S1 Fig). To control for ebolavirus cross-reactivity [23], a separate cutoff was implemented at the three-fold change above the arithmetic mean of the mock-adjusted scaled MFI. Positive samples were defined as exceeding both thresholds. The 2018 Bio-Plex data was analysed with the same two statistical methods, but not mock-adjusted (S2 Fig). The spread of MFI values for duplicate measurements of each individual sample was plotted (S3 and S4 Figs). Mean negative human sera MFI values were plotted and the standard deviation for each sample was presented (S5 Fig). All analyses and visualizations were implemented in R 3.5.1 [24]; the R code can be retrieved from the authors upon request.

## Results

**Fig 2:**
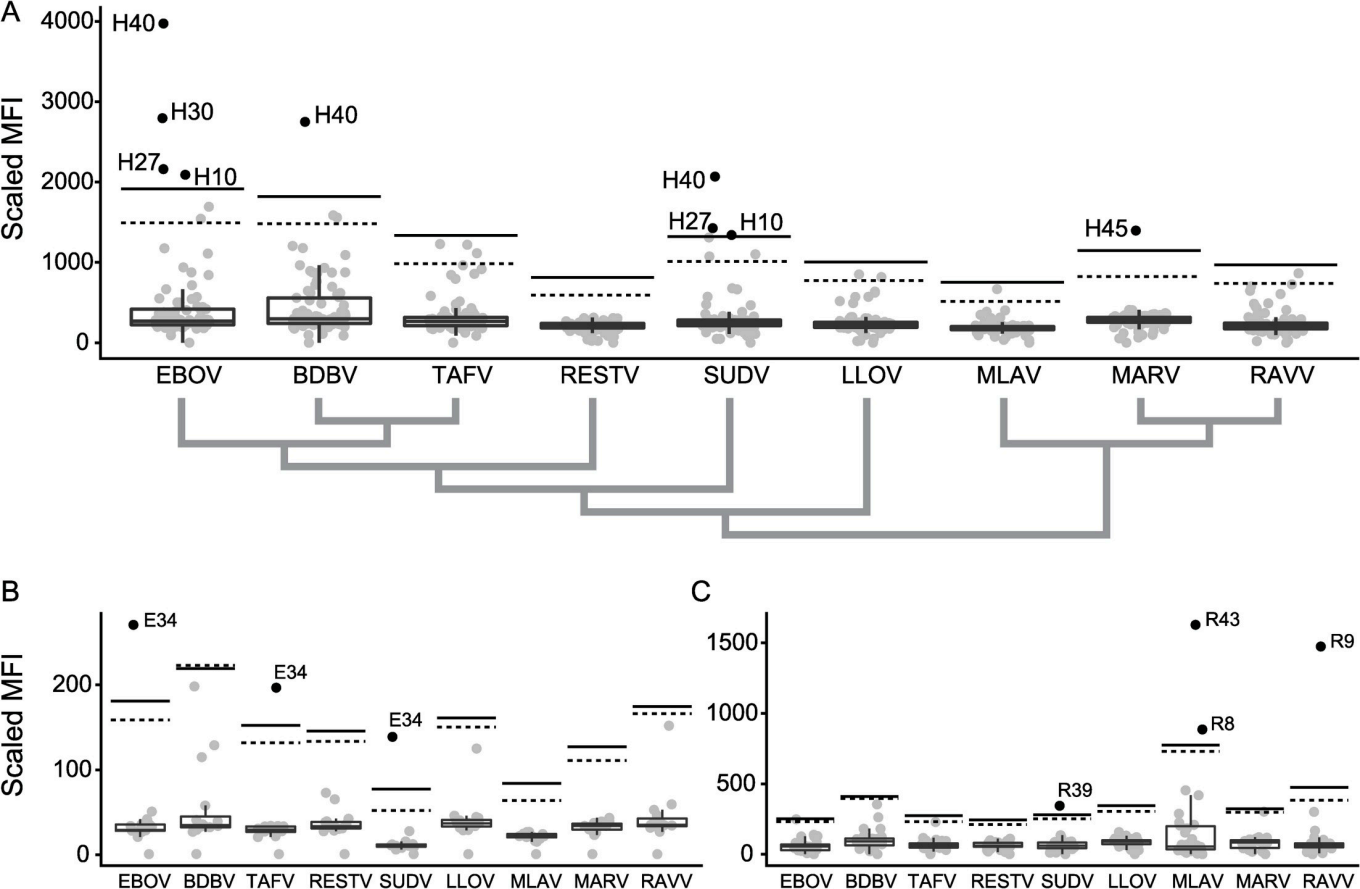
MFI values for sera obtained from humans (A), *Eonycteris spelaea* (B), *Rousettus leschenaultii* (C). Antibodies reactive to filovirus GPe from Ebola virus (EBOV), Bundibugyo virus (BDBV), Taï Forest virus (TAFV), Sudan ebolavirus (SUDV), Reston virus (RESTV), Lloviu virus (LLOV), Měnglà virus (MLAV), Marburg virus (MARV), and Ravn virus (RAVV) are quantified in a bead-based fluorescence assay. Grey dots represent individual samples. A boxplot is overlaid to indicate median, quartiles and extremes of the sample distribution. A black dashed line indicates the cutoff determined from a single lognormal curve-fit and a black solid black line the three-fold increase over the mean. A cladogram in panel A indicates the phylogenetic relationships of individual filovirus GPe based on their amino acid sequence.

The majority of bat hunters were between 18 and 50 years of age, male, and participated at least eleven times in the harvest (Table 2). All bat tissues tested were PCR-negative for filovirus-specific nucleic acid. There were a total of 13,993,300 reads from the *R*. *leschenaultii* NGS dataset and 7,975,905 reads from the *E*. *spelaea* NGS dataset and no filovirus sequences were identified. In our 2019 serum screen (that included MLAV), we detected filovirus-reactive sera in 5.9% (5/85) of human samples, 6.2% (1/16) of *E*. *spelaea* samples, and 13.3% (4/30) of *R*. *leschenaultii* samples. The highest MFI values, corresponding to levels of filovirus-specific serum IgG, were detected for EBOV-GPe in human and *E*. *spelaea* sera, and for MLAV- and RAVV-GPe in *R*. *leschenaultii* sera (Fig 2). Our results suggest human exposure to two antigenically distinct filoviruses, the first group of sera (H10, H27, H30, H40) being reactive to EBOV-, BDBV-, and SUDV-GPe, and one individual serum (H45) singly reactive to MARV-GPe. An individual *E*. *spelaea* serum sample (E34) reacted to the EBOV-, SUDV- and TAFV-GPe, displaying a similar cross-reactivity pattern as seen for the first group of human sera and was previously reported for filovirus-positive *E*. *spelaea* samples from Singapore [7]. The 2019 screening results corroborated positive samples that were detected when screened in 2018 using a Bio-Plex 200 system (S4 Fig; H27, H30, H40, E34, R39), strengthening interpretation of positive sera samples screened in two different years with two different Luminex xMAP-based machines. There was minimal intra- and inter-individual variation in the negative human sera samples and these were all well below the MFI cutoff values for each GPe (S5 Fig).

**Table 2:** Basic demographic information on human study population.

Population		Age distribution		Participation in bat harvest	
Individuals	85 (100%)	18–30 years	36 (42.4%)	0–10 times	25 (29.4%)
Male	50 (58.8%)	31–50 years	36 (42.4%)	11–25 times	40 (47.1%)
Female	35 (41.2%)	≥51 years	13 (15.3%)	≥ 26 times	20 (23.5%)

## Discussion

Despite the growing evidence that filoviruses are present in South and Southeast Asia, there has been a historical absence of outbreaks of filovirus haemorrhagic fever in this region. Reasons why clusters of human filovirus infections have not yet been detected in Asia, include (a) ecological barriers prevent zoonotic transmission, (b) viruses are unable to sustain transmission between humans or (c) an uncharacterized diversity of non-pathogenic, antigenically-related filoviruses exist and cause asymptomatic infection in humans. Human populations with wildlife contact and no history of Ebola virus disease in Uganda [25] and the Democratic Republic of Congo [26] were reportedly ebolavirus seropositive. Similarly, here we report the presence of filovirus (e.g. ebolavirus, marburgvirus and dianlovirus) reactive antibodies in both human (e.g. bat hunters) and bat populations in Northeast India, a region with no historical record of Ebola virus disease.

Cross-reactivity between EBOV, BDBV and SUDV of the tested samples is in agreement with a previous report [23]. The mammalian cell-culture expression system to produce oligomeric, native-like GPe capture antigens in this multiplex assay provides several benefits compared to peptide-based antigen assays, including the retention of post-translational modifications (i.e. glycosylation) and native quaternary structures allowing capture of conformational-dependent antibodies. The use of GPe from all presently described filoviruses—with the exception of the recently described Bombali virus—allows for simultaneous detection and antigenic differentiation of virus species-specific IgGs and the identification of cross-reactive IgG responses. Most ebolavirus serology surveillance studies are unable to address the complex biology of known and unknown filoviruses in terms of cross-reactivity of specific antibodies. This filovirus serological assay addresses many limitations of previously employed assays by using oligomeric, native-like virus antigens in a multiplex manner and represents an improved biosurveillance tool. Establishing thresholds for low sample number serum sets that lack both positive and negative controls is challenging, but our efforts to employ two independent statistical methods and two machine platforms yielded congruent results and is in agreement with prior approaches to estimate seropositivity [27, 28].

Furthermore, this study describes serum reactivity to MLAV, incidentally in the same bat species (*R*. *leschenaultii*) mentioned in the initial report [2]. Reactivity to RESTV, which circulates endemically among bats, pigs and monkeys in the Philippines [29], and causes subclinical infection in animal care takers and slaughterhouse workers in the Philippines [9], was not detected in our study. Interestingly, MLAV and RAVV positive *R*. *leschenaultii* sera suggest circulation of two distinct filoviruses within the same species, which is serologically distinct from reactivity with *E*. *spelaea* and human samples. These results are concordant with previous findings of exposure to filoviruses antigenically closely related to EBOV, BDBV, and SUDV in *E*. *spelaea* in Singapore [17].

Two proposed mechanisms for sustained virus infection in the studied bat species are frequent co-roosting with other bats and the introduction of large numbers of susceptible juveniles into the population [30]. The two bat species sampled in this study, *R*. *leschenaultii* and *E*. *spelaea*, roost in large colonies in caves with rolling parturition patterns [31]. Though we have serological evidence of filovirus exposure, there was no genomic data detected. There are several reasons why this may be, including; small sample size, low virus copy numbers, uncertain epidemiological shedding periodicity, and high filovirus genetic diversity that is not captured by the primers employed here. Our results reinforce the need to select sentinel sites for virus surveillance at the human-animal interface and highlights some of the gaps in our understanding of filovirus transmission and ecology.

## Supporting information

S1 Appendix Technical description of next generation sequencing sample preparation, library preparation, sequencing, and bioinformatic pipeline. (DOCX)

S1 Fig Histograms for each GPe (Ebola virus (EBOV), Bundibugyo virus (BDBV), Taï Forest virus (TAFV), Sudan virus (SUDV), Reston virus (RESTV), Lloviu virus (LLOV), Marburg virus (MARV), and Ravn virus (RAVV), Měnglà virus (MLAV) and mock antigen (MOCK)) and sera from humans (A), *Eonycteris spelaea* (B), and *Rousettus leschenaultii* (C). Lognormal distribution representing the best fit of all samples are indicated by solid black lines. A solid black vertical lines indicates 3-fold over mean and a dotted black line denotes cutoff established by lognormal curve fitting.
(TIF)

S2 Fig MFI values for sera obtained from humans (A), *Eonycteris spelaea* (B), *Rousettus leschenaultii* (C) screened in 2018 on a Bio-Plex machine. Antibodies reactive to filovirus GPe from Ebola virus (EBOV), Bundibugyo virus (BDBV), Taï Forest virus (TAFV), Sudan ebolavirus (SUDV), Reston virus (RESTV), Lloviu virus (LLOV), Marburg virus (MARV), and Ravn virus (RAVV) are quantified in a bead-based fluorescence assay. Grey dots represent individual samples. A boxplot is overlaid to indicate median, quartiles and extremes of the sample distribution. A black dashed line indicates the cutoff determined from a single lognormal curve-fit and a black solid black line the three-fold increase over the mean.
(TIF)

S3 Fig For the 2018 Bio-Plex dataset, mean values (horizontal lines) and spread of the two individual measurements are shown (vertical lines) for sera from human (A), *Eonycteris spelaea* (B) and *Rousettus leschenaultii* (C).
(TIF)

S4 Fig For the 2019 MAGPIX dataset, mean values (horizontal lines) and spread of the two individual measurements are shown (vertical lines) for sera from *Eonycteris spelaea* (A) and *Rousettus leschenaultii* (B).
(TIF)

S5 Fig Mean values and standard deviation of seven normal human serum samples from healthy volunteers (varying colours). Samples were tested in our assay with the indicated antigens in eight technical replicates.
(TIF)

S1 Table Raw MFI values for human samples diluted at 1:100. (XLSX)

S2 Table Raw MFI values for *Eonycteris spelaea* samples diluted at 1:250. (XLSX)

S3 Table Raw MFI values for *Rousettus leschenaultii* samples diluted at 1:250. (XLSX)

S4 Table Negative human sera control values for seven individuals with eight technical replicates. (XLSX)
