# Correction: Filovirus-reactive antibodies in humans and bats in Northeast India imply zoonotic spillover

**DOI:** 10.1371/journal.pntd.0009836

**Published:** 2021-11-16

**Authors:** Pilot Dovih, Eric D. Laing, Yihui Chen, Dolyce H. W. Low, B. R. Ansil, Xinglou Yang, Zhengli Shi, Christopher C. Broder, Gavin J. D. Smith, Martin Linster, Uma Ramakrishnan, Ian H. Mendenhall

There is an error in the affiliations for author BR Ansil. Manipal Academy of Higher Education, Manipal, Karnataka, India, should not be listed. The correct affiliation for BR Ansil is as follows: National Centre for Biological Sciences, Tata Institute of Fundamental Research, Bangalore, India.

Additional information is provided regarding study methodology. The anonymity of human participants was maintained throughout the study. No live animals were handled or euthanized for the purpose of this study and only dead bats collected from villagers following an annual community event were used.

The authors confirm that the study received all necessary approvals from the necessary national statutory bodies. The following permits and approvals were obtained:

- A land use permit from Government of Nagaland, Office of the Additional Deputy Commissioner, Pungro, Nagaland, India;
- Security and sensitivity clearance of the competent authority, Department of Atomic Energy, the parent funding body of National Centre for Biological Sciences, Tata Institute of Fundamental Research (NCBS-TIFR);
- Permission from the Bomrr Clan for research at the privately owned study site;
- Approval for an exemption from the National University of Singapore (NUS) Institutional Review Board;
- Approval from the NUS Institutional Animal Care and Use Committee;
- Approval from the National Centre for Biological Sciences (NCBS) Institutional Ethics Committee (Human Subjects);
- Approval from the NCBS Institutional Animal Ethics Committee;
- Approval from the NCBS Institutional Biosafety Committee.

Copies of documentation for the above permits and approvals have been provided to the journal editors.

At the time of publication of this notice, PLOS has been unable to reach the Indian Council of Medical Research to discuss the government-level regulations and permits relevant to this study. However, the Director of NCBS-TIFR confirmed to the journal editors that this study complied with all local regulations and received all necessary permissions, permits and had necessary approval of the parent Department i.e. the Department of Atomic Energy, Government of India.
