# Supplementary material for: Filovirus-reactive antibodies in humans and bats in Northeast India imply zoonotic spillover
Source: PLoS Negl Trop Dis. 2019 Oct 31;13(10):e0007733. doi: 10.1371/journal.pntd.0007733 (PMC6822707; doi:10.1371/journal.pntd.0007733)
Supplement: S1 Appendix — (DOCX) [file pntd.0007733.s001.docx]

**Technical Appendix for:**

Filovirus-reactive antibodies in humans and bats in Northeast India imply zoonotic spillover

Pilot Dovih^1,2^, Eric D. Laing^3^, Yihui Chen^4^, Dolyce H.W. Low^4,5^, B.R Ansil^1^, Xinglou Yang^6^, Zhengli Shi^6^, Christopher C. Broder^3^, Gavin J.D. Smith^4^, Martin Linster^4^, Uma Ramakrishnan^1^, Ian H. Mendenhall^4^*

**Next generation sequencing library preparation and sequence analysis**

Kidney, lung and spleen tissues were collected from *E. spelaea* (n=34) and *R. leschenaultii* (n=34) during the bat harvest and stored in RNA*later*® (Sigma-Aldrich) for downstream processing at the NCBS research institute. 1mm^3^ of each tissue was excised, combined by individual and homogenized in AVL Buffer (Qiagen) in a biosafety level 2 biosafety cabinet. An aliquot of homogenate was used in downstream PCR analysis, while another aliquot of homogenate was used for NGS analysis.

Aliquots of homogenate for NGS analysis were further pooled by species (*E. spelaea* = 34 individuals and *R. leschenaultii* = 34 individuals). 200μL of pooled homogenate was diluted with 520uL of 1X PBS and divided into four equal 180μL aliquots. Each aliquot was subsequently filtered through a 0.45μm and a 0.22μm cellulose acetate spin filter. 150μL of the filtrate was treated by an enzyme mix adapted from Hansen *et al*., 2016 (1). 14μL Turbo DNase (AM1907, Ambion, ThermoFisher Scientific), 6μL Baseline ZERO™ DNase (DB0715K, Epicentre, Lucigen Corporation), 6μL RNase Cocktail™ Enzyme Mix (AM2286, Ambion, ThermoFisher Scientific), 20.5μL 10X Turbo DNase Buffer, and sterile water to top up to 205μL total reaction volume was incubated for two hours at 37°C. Enzyme-treated RNA was pooled and extracted using the Direct-zol™ RNA Mini Prep kit (Zymo Research) according to manufacturer’s instruction.

Extracted viral RNA was converted to cDNA as described by Lewandowska *et al*., (2017) (2). Firstly, first strand cDNA was synthesized by incubating 10pg-5μg of RNA in a 13μL reaction containing 0.77mM dNTPs and 7.7μM Anchor primer (5’ – ATCGTCGTCGTAGGCTGCTCNNNNNNNN – 3’) at 65°C for 5 mins and ice for 1 min. A 20μL reaction containing 13μL of the first reaction, 1μl SuperScript™ III Reverse Transcriptase (18080-044, Invitrogen, ThermoFisher Scientific), 1X First Strand Buffer, 5μM DTT and 1μl RNaseOUT™ Recombinant Ribonuclease Inhibitor (10777-019, Invitrogen, ThermoFisher Scientific) was incubated at 42°C for 1 hour and 72°C for 15 mins. Second strand cDNA was synthesized in a 20μL reaction by adding 2-5μg first strand cDNA with 125μM dNTP, 1μl DNA polymerase I, Large (Klenow) Fragment (M0210L, NEB), 1X NEBuffer™ 2 (B7002S, NEB), and incubated at 37°C for 30 mins, followed by 75°C for 20 mins. A 20μL reaction containing 5μg DNA and 5μM Anchor primer was denatured at 94°C for 2 mins and 10°C for 10 mins. Second strand DNA was synthesized as described with second strand cDNA. A final DNA amplification step was performed by preparing a 50μL reaction consisting of 1μg dsDNA, 0.25μl AmpliTaq Gold™ DNA Polymerase (4311818, Applied Biosystems™, ThermoFisher Scientific), 1X PCR Gold Buffer, 75mM MgCl_2_, 40mM dNTPs, and 50μM Anchor primer. The amplification was performed with an initial denaturation at 94°C for 15 mins, 40 cycles of denaturation at 94°C for 30 secs, annealing at 40°C and 50°C for 30 secs each, extension at 72°C for 1 min, and a final extension at 72°C for 5 mins. The PCR product was purified with 1.8X Mag-Bind® RxnPure Plus (Omega Bio-tek) magnetic beads. NGS libraries were prepared with Illumina Nextera XT DNA Library Prep Kit (FC-131-1024, Illumina Incorporation), validated on a Bioanalyzer machine and sequenced on a HiiSeqX Illumina machine with 2x150bp reads by Medgenome Labs Ltd (Bangalore, India).

All FASTQ files were examined using FastQC to assess overall quality (3). Trimming was performed using Trimmomatic 0.3.2 to remove adapters, low quality bases (Q=20 and reads with fewer than 50bp in length (4). Read classification was performed with DIAMOND and sequence similarity searches were executed against a local National Center for Biotechnology Information (NCBI) non-redundant protein database (5). DIAMOND outputs were imported into MEGAN6 for the taxonomic assignment of reads and visualizing of the distribution of virus sequences (6).

1. Hansen TA, Mollerup S, Nguyen NP, White NE, Coghlan M, Alquezar-Planas DE, et al. High diversity of picornaviruses in rats from different continents revealed by deep sequencing. Emerg Microbes Infect. 2016;5(8):e90.

2. Lewandowska DW, Zagordi O, Geissberger F-D, Kufner V, Schmutz S, Böni J, et al. Optimization and validation of sample preparation for metagenomic sequencing of viruses in clinical samples. Microbiome. 2017;5(1):94.

3. Andrews S. FastQC 2016 [Available from: <http://www.bioinformatics.bbsrc.ac.uk/projects/fastqc/>.

4. Bolger AM, Lohse M, Usadel B. Trimmomatic: a flexible trimmer for Illumina sequence data. Bioinformatics (Oxford, England). 2014;30(15):2114-20.

5. Buchfink B, Xie C, Huson DH. Fast and sensitive protein alignment using DIAMOND. Nat Methods. 2015;12(1):59-60.

6. Huson DH, Beier S, Flade I, Gorska A, El-Hadidi M, Mitra S, et al. MEGAN Community Edition - Interactive Exploration and Analysis of Large-Scale Microbiome Sequencing Data. PLoS Comput Biol. 2016;12(6):e1004957.
