# Supplementary figures and images for: Filovirus-reactive antibodies in humans and bats in Northeast India imply zoonotic spillover
Source: PLoS Negl Trop Dis. 2019 Oct 31;13(10):e0007733. doi: 10.1371/journal.pntd.0007733 (PMC6822707; doi:10.1371/journal.pntd.0007733)

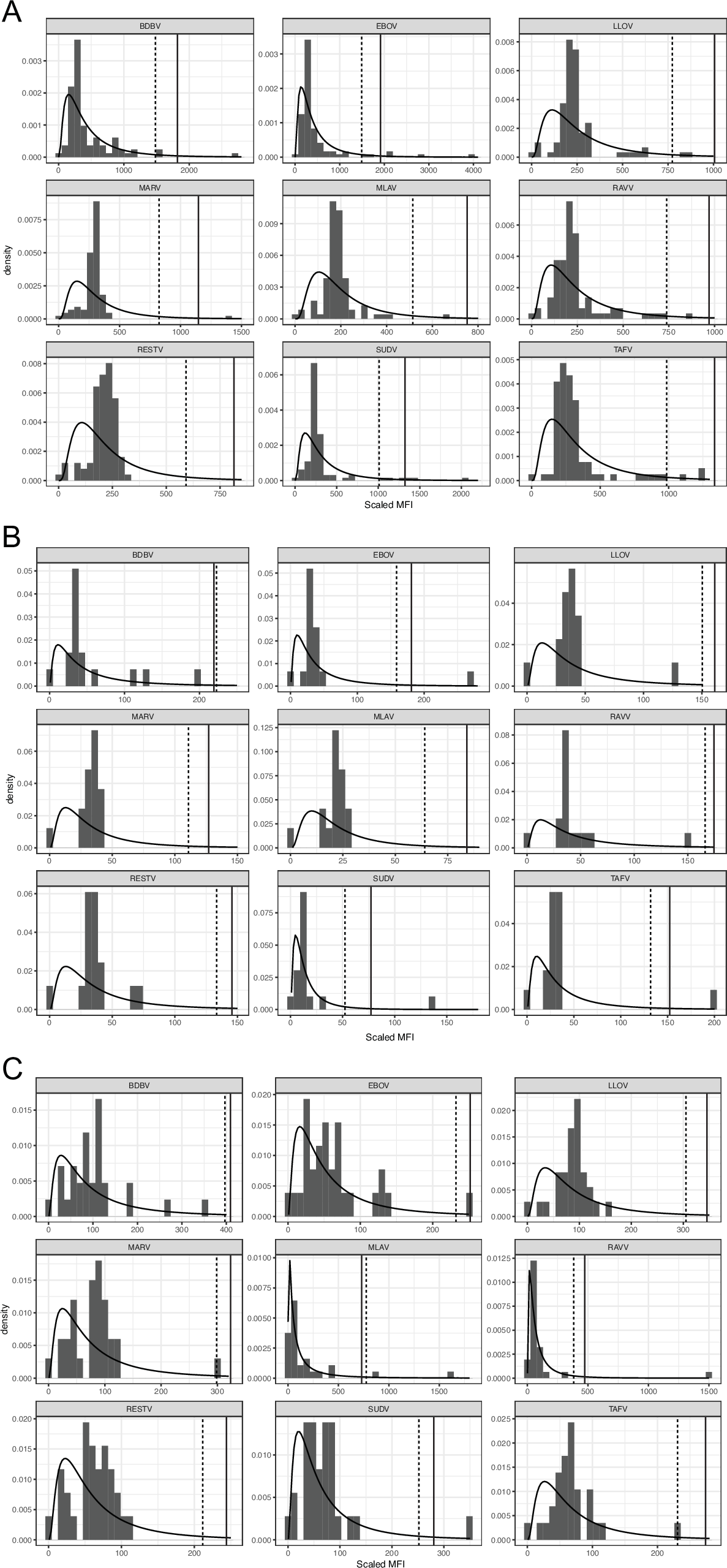

Supplement: S1 Fig — Histograms for each GPe (Ebola virus (EBOV), Bundibugyo virus (BDBV), Taï Forest virus (TAFV), Sudan virus (SUDV), Reston virus (RESTV), Lloviu virus (LLOV), Marburg virus (MARV), and Ravn virus (RAVV), Měnglà virus (MLAV) and mock antigen (MOCK)) and sera from humans (A), Eonycteris spelaea (B), and Rousettus leschenaultii (C). Lognormal distribution representing the best fit of all samples are indicated by solid black lines. A solid black vertical lines indicates 3-fold over mean and a dotted black line denotes cutoff established by lognormal curve fitting. (TIF) [file pntd.0007733.s002.tif]

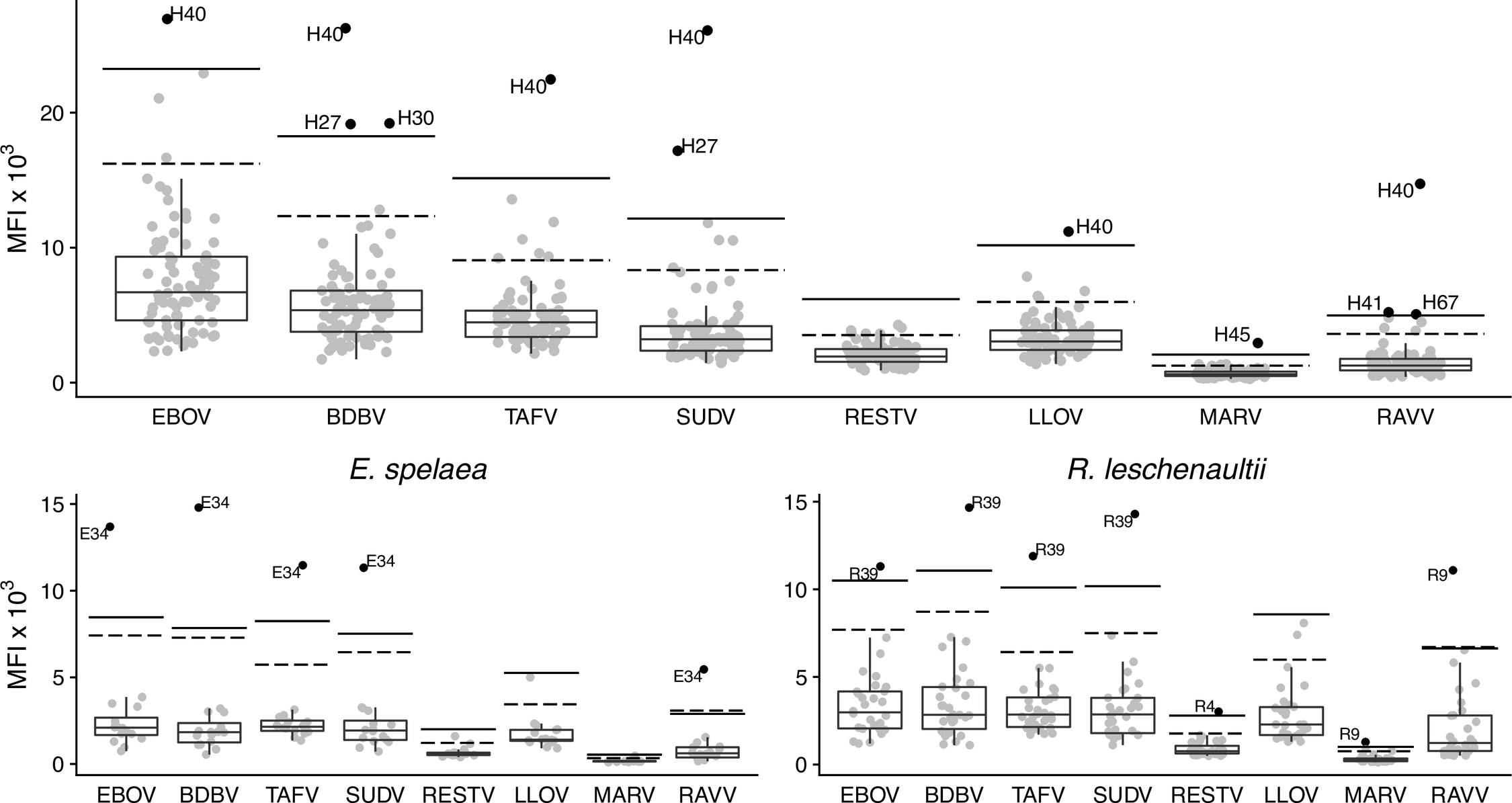

Supplement: S2 Fig — MFI values for sera obtained from humans (A), Eonycteris spelaea (B), Rousettus leschenaultii (C) screened in 2018 on a Bio-Plex machine. Antibodies reactive to filovirus GPe from Ebola virus (EBOV), Bundibugyo virus (BDBV), Taï Forest virus (TAFV), Sudan ebolavirus (SUDV), Reston virus (RESTV), Lloviu virus (LLOV), Marburg virus (MARV), and Ravn virus (RAVV) are quantified in a bead-based fluorescence assay. Grey dots represent individual samples. A boxplot is overlaid to indicate median, quartiles and extremes of the sample distribution. A black dashed line indicates the cutoff determined from a single lognormal curve-fit and a black solid black line the three-fold increase over the mean. (TIF) [file pntd.0007733.s003.tif]

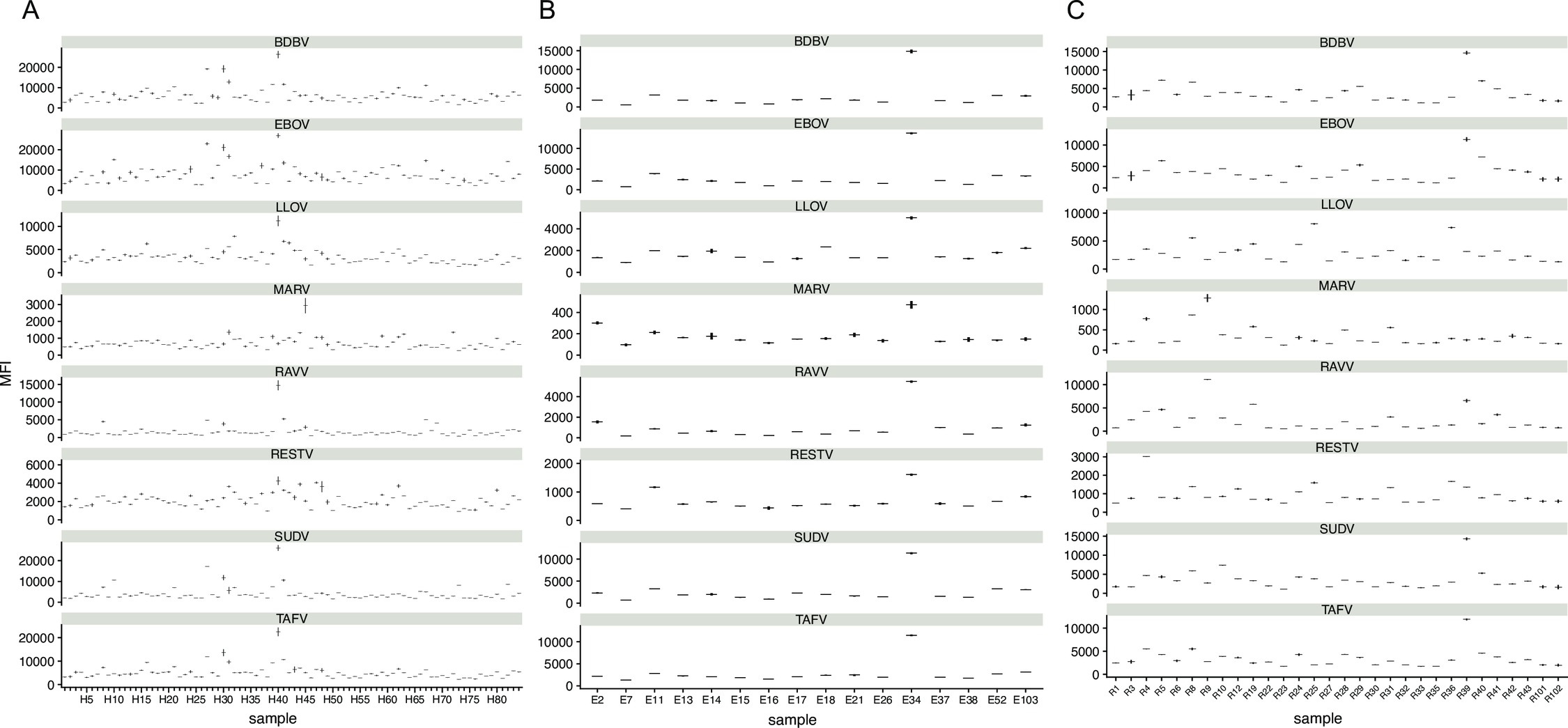

Supplement: S3 Fig — For the 2018 Bio-Plex dataset, mean values (horizontal lines) and spread of the two individual measurements are shown (vertical lines) for sera from human (A), Eonycteris spelaea (B) and Rousettus leschenaultii (C). (TIF) [file pntd.0007733.s004.tif]

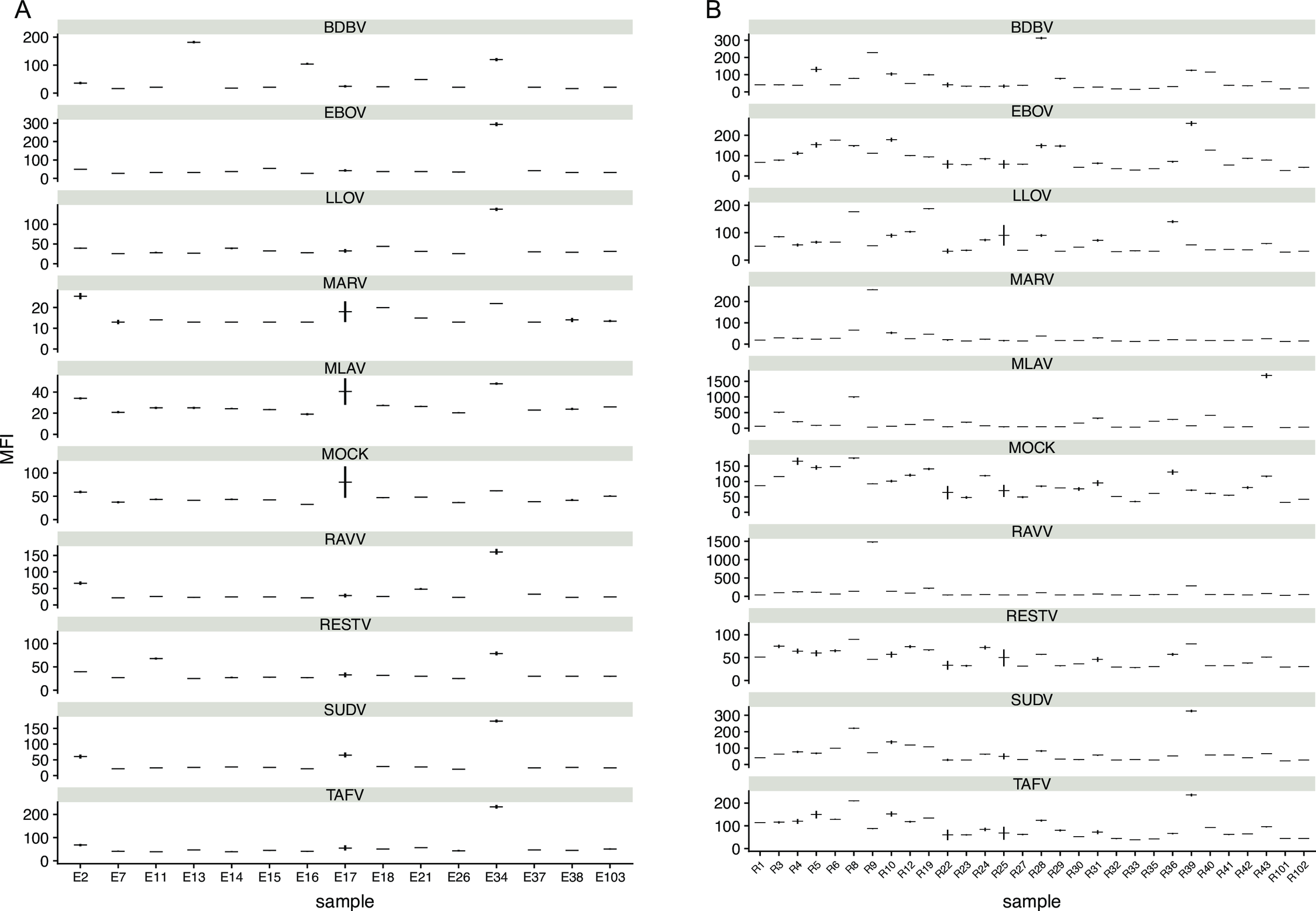

Supplement: S4 Fig — For the 2019 MAGPIX dataset, mean values (horizontal lines) and spread of the two individual measurements are shown (vertical lines) for sera from Eonycteris spelaea (A) and Rousettus leschenaultii (B). (TIF) [file pntd.0007733.s005.tif]

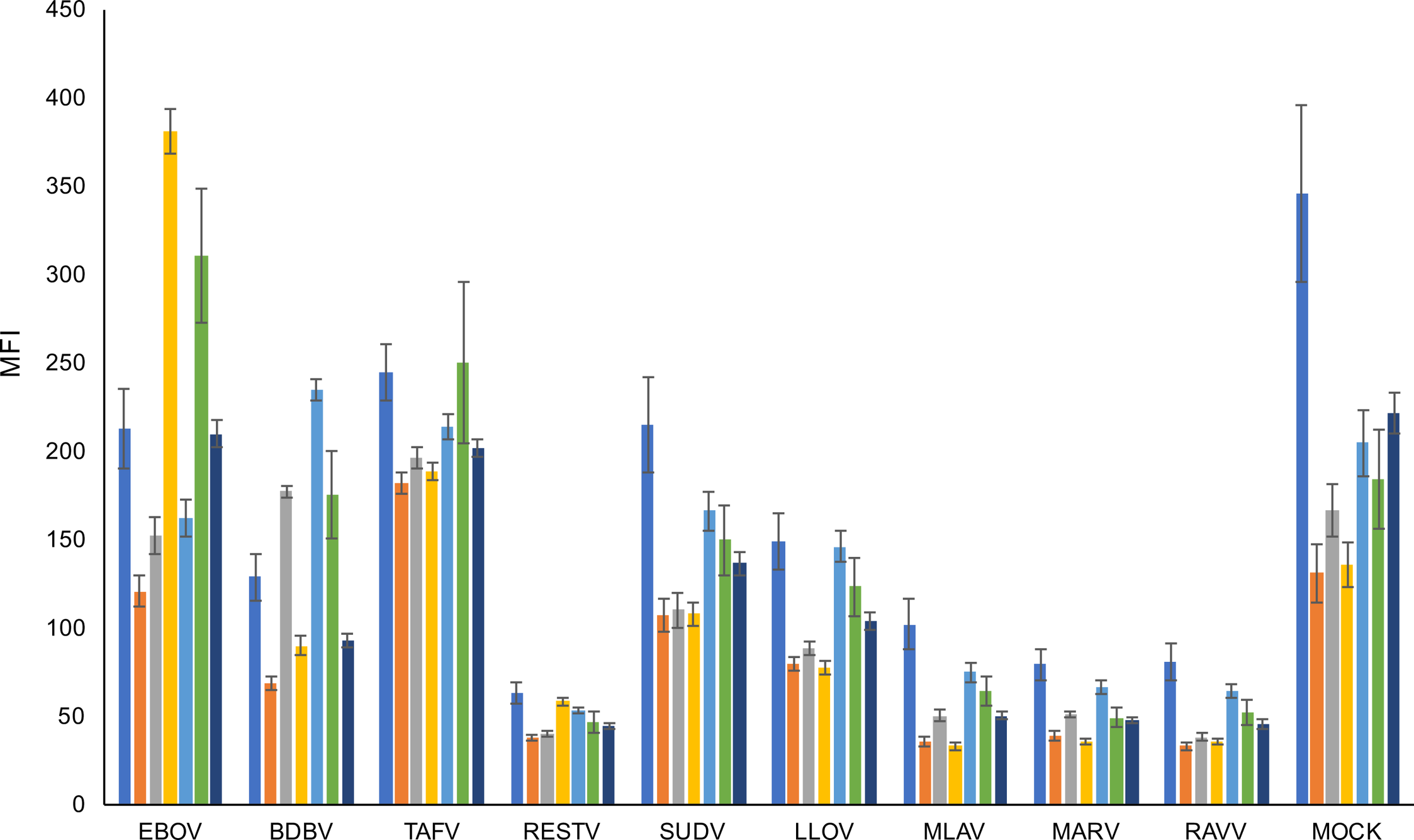

Supplement: S5 Fig — Samples were tested in our assay with the indicated antigens in eight technical replicates. (TIF) [file pntd.0007733.s006.tif]
